# Joint count transformation models with covariate‐dependent correlations

**DOI:** 10.1002/ecy.70522

**Published:** 2026-10-12

**Authors:** Lukas Graz, Luisa Barbanti, Roland Brandl, Torsten Hothorn

**Affiliations:** ^1^ Epidemiology, Biostatistics and Prevention Institute University of Zurich Zürich Switzerland; ^2^ Department of Biology University of Marburg Marburg Hessen Germany

**Keywords:** abundance data, count data, covariate‐dependent Gaussian copula, distribution‐free models, Gaussian copula, interspecific correlation, joint species distribution models, transformation models

## Abstract

Joint species distribution models are essential for understanding how ecological covariates shape species communities. However, most existing approaches are limited by rigid parametric distributions for count data and the inability to model how interspecific associations change with those covariates. We introduce joint count transformation models, a novel framework designed to overcome these limitations. Our approach combines distribution‐free marginal count transformation models for multiple species with a covariate‐dependent latent Gaussian copula to model interspecific correlations, interpretable as Spearman's rank correlation on the observed count scale. All model parameters are estimated efficiently via joint maximum likelihood estimation, implemented in the R package *tram*. We apply this framework to model the joint abundance of three fish‐eating bird species, using seasonality as the primary covariate. Our model successfully captured the complex, species‐specific seasonal abundance patterns, including periods of high zero counts and seasonal shifts in variance. Furthermore, the model revealed strong, seasonally varying correlations between the species. These findings are consistent with an empirical approach and similar to those from the computationally expensive parametric Bayesian Hierarchical Modeling of Species Communities (HMSC) framework. Consistency, accuracy, and feasibility of our approach are demonstrated in a simulation study for up to 10 species.

## INTRODUCTION

Ecology studies how individual species and community interactions—spanning from mutualism and commensalism to predation and competition—are governed by a range of covariates, such as physical habitat, climatic conditions, and the broader spatiotemporal context. Species distribution models (SDMs) have become popular tools for describing the relationship of these covariates with the distribution of a species' occurrence or abundance. Predictions from SDMs are valuable not only in fundamental ecological research but also in applied ecology, especially for the conservation of endangered species and the management of invasive ones. Binary regression models and classification machine learning methods are often used to predict the presence or absence of a species in a given habitat (e.g., Cowans et al., 2025; Elith et al., 2008; Hothorn et al., 2011). Models describing species abundance rely on count data instead of presence/absence information and thus yield more detailed insights into a species' distribution.

Standard SDMs inherently ignore the interspecific relationships within an ecological community. To address this limitation, and building upon early work in community‐level modeling (De'ath, 2002), more elaborate models that integrate both community and environmental aspects have been proposed over the past decade. Early ad hoc approaches included incorporating the presence or absence of one species as a predictor variable in the SDM for another (Kissling et al., 2012). Seeking to better disentangle shared environmental preferences from true biotic interactions, the field shifted toward more sophisticated model‐based approaches. Ingram et al. (2020) categorized these into multispecies distribution models (MSDMs)—which model multiple species simultaneously to share information across their environmental responses (e.g., Ovaskainen & Soininen, 2011), sometimes utilizing a priori structured correlations to encode phylogenetic information (Ives & Helmus, 2011)—and joint species distribution models (JSDMs).

Conversely, JSDMs are defined as statistical frameworks that simultaneously model the joint distribution of multiple species while explicitly accounting for both shared environmental responses and residual correlations between species co‐occurrence patterns. These approaches collectively rely on multivariate generalized linear mixed models (MGLMMs) and latent variable models to explicitly capture community dynamics (e.g., Ovaskainen et al., 2010; Pollock et al., 2014; Warton et al., 2015). While JSDMs typically rely on unstructured residual correlations, recent advancements have also introduced structured correlations to improve the modeling of these interspecific dependencies (Heaps & Jermyn, 2024). Despite these conceptual advancements, the modeling approaches implemented by MSDMs and JSDMs remain largely mutually exclusive (see Norberg et al., 2019, for an empirical comparison): MSDMs estimate interpretable marginal models for each species but lack an explicit assessment of their relationships, whereas JSDMs allow the identification of species relationships without providing interpretable marginal models.

This motivates a model in which the dependence structure itself varies with covariates rather than being treated as a static residual term. In the following, we therefore focus on a covariate‐dependent copula framework that can retain interpretable marginal SDMs while allowing pairwise associations to change over time or across other environmental gradients. In econometrics, time‐varying copulas are used to estimate the correlation of different asset values depending on time in an autoregressive manner (Patton, 2006). A key problem is that they are designed for continuous time series, limiting their applicability to count data and covariates other than time. Within ecology, studies such as Bakker et al. (2006) or Petren and Case (1998) have shown that competition/correlation patterns may also change in response to changes in environmental conditions. However, most modern JSDMs are MGLMMs or generalized linear latent variable models (GLLVMs), such as those introduced by McGillycuddy et al. (2025) and Niku et al. (2019). Neither MGLMMs nor GLLVMs allow for covariate‐dependent correlations. Hui et al. (2023) extended MGLMMs by adding a spatiotemporal (spline‐based) random effect, where correlation is spatially (and potentially temporally) structured. Han and De Oliveira (2020) introduced Gaussian copula models for geostatistical count data, and Anderson et al. (2019) and Popovic et al. (2018) modeled multivariate correlated counts via a latent Gaussian copula, but none of these approaches allow for covariate‐dependent correlations. Furthermore, instead of estimating all parameters jointly, these copula‐based methods rely on a two‐step procedure—estimating the marginals before the static copula parameters—which leads to incorrect SEs. Conditional graphical LASSO (Augugliaro et al., 2023) estimates the copula and marginals jointly but is limited to static correlations and cannot accommodate count data.

A relevant extension to MGLMMs was developed by Tikhonov et al. (2017) within the Hierarchical Modeling of Species Communities (HMSC) framework, which does allow for covariate‐dependent correlations. This model class was identified as the best performing approach in a large‐scale benchmark comparison of 33 JSDMs (Norberg et al., 2019), making it the state‐of‐the‐art competitor for our method. However, the HMSC implementation is restricted to (log‐)Poisson distributed counts and comes with the usual caveats of Bayesian Markov chain Monte Carlo (MCMC) estimation, such as long computation times and potential convergence issues.

In the following, we present joint count transformation models (JCTMs), a novel and flexible JSDM framework designed to overcome these limitations by estimating the marginal distributions and covariate‐dependent correlations in a data‐driven, distribution‐free manner. Our approach is built upon marginal SDMs and a covariate‐dependent copula connecting them. Standard SDMs typically rely on rigid parametric distributions (e.g., Poisson or negative binomial) or apply arbitrary a priori transformations (e.g., $\log\left(y+1\right)$) that incorrectly assume continuous likelihoods for discrete data. To resolve this, our marginal SDMs employ the count transformation models of Siegfried and Hothorn (2020). This distribution‐free framework estimates the data transformation simultaneously with covariate effects by maximizing the exact discrete log‐likelihood, naturally accommodating complex features like over‐ and underdispersion. We extend this foundation to the location‐scale transformation models of Siegfried et al. (2023), allowing both the location and scale of the transformed counts to depend on covariates. The structure of the JCTM emerges from multivariate conditional transformation models (Klein et al., 2022), in which the correlation structure is described by a covariate‐dependent latent Gaussian copula. Joint maximum likelihood estimation and inference for all parameters is performed using the appropriate count likelihood and its gradient from Hothorn (2026a, 2026b) implemented in Hothorn (2026c).

One could obtain marginal SDMs from commonly applied JSDMs (i.e., MGLMMs and GLLVMs) by integrating over the species‐random effects, but the simple interpretation of parameters in linear predictors is lost in the process (Lee & Nelder, 2004; Muff et al., 2016). For JCTMs, however, marginal SDMs are directly available as a by‐product of the model fitting procedure.

The section *Joint count transformation models* formally introduces JCTMs. An example application of a JCTM is provided in the *Case study: Fish‐eating aquatic birds*, presenting marginal distributions (i.e., SDMs) and comparing model‐based correlation curves with an empirical approach and HMSC. The results are examined in the *Discussion*, along with potential directions for future work. A simulation study investigating how feasibility, consistency and accuracy of JCTMs change with sample size and the number of species is provided in Appendix S1.

## JOINT COUNT TRANSFORMATION MODELS

Our approach extends Multivariate Conditional Transformation Models, originally proposed by Klein et al. (2022), to multivariate count responses. JCTMs model the joint distribution of J species count variables $Y=\left(Y_{1},\ldots ,Y_{J}\right)\in {{\mathbb{N}}_{0}}^{J}$ conditional on a set of covariates x. Hereafter, the subscripts j and ȷ refer to any of the 1,…,J species.

### General model structure

We combine the individual SDMs introduced in *Marginal species distribution models* into a JSDM leveraging a latent Gaussian copula $\Phi_{0,\Sigma \left(x\right)}$, that is, using the cumulative distribution function of a multivariate normal distribution with a zero‐mean vector and a covariate‐dependent correlation matrix $\Sigma \left(x\right)$ (i.e., constraining $\Sigma {\left(x\right)}_{\mathrm{jj}}=1$ in *Covariate‐dependent correlation*). The JCTM is defined by the joint cumulative distribution of Y conditional on X=x as

(1) $$\mathbb{P}\left(Y\leq y|X=x\right)=\Phi_{0,\Sigma \left(x\right)}\left(h\left(y|x\right)\right),$$

where we call $h\left(y|x\right)={\left(h_{1}\left(y_{1}|x\right),\ldots ,h_{J}\left(y_{J}|x\right)\right)}^{⊤}$ a conditional transformation function. In order to yield a valid discrete multivariate distribution for count data, $h_{j}\left(\cdot |x\right)$ needs to be monotonically increasing (for all x) and constant on the interval $\left[k-1,k\right)$ for any integer k∈ℕ to ensure that the model assigns probabilities only to integer counts. Furthermore, the transformation of $y_{j}$ may depend on covariates x, enabling covariate‐dependent shifts, scaling, and stratification of the response distribution.

### Marginal species distribution models

A key feature of the Gaussian copula framework is that the joint model in Equation 1 decomposes into separate marginal distributions, which serve as the individual SDMs for each species:

(2) $$\mathbb{P}\left(Y_{j}\leq y_{j}|X=x\right)=\Phi_{0,1}\left(h_{j}\left(y_{j}|x\right)\right).$$

The conditional probability mass function for an observed count $y_{j}\in {\mathbb{N}}_{0}$ of species j is then

(3) $$p_{Y_{j}|X=x}\left(y_{j}\right)=\left\{\begin{array}{ll} \Phi_{0,1}\left(h_{j}\left(y_{j}|x\right)\right)-\Phi_{0,1}\left(h_{j}\left(y_{j}-1|x\right)\right), & \text{if}\;y_{j}=1,2,...\\ \Phi_{0,1}\left(h_{j}\left(y_{j}|x\right)\right), & \text{if}\;y_{j}=0 \end{array}\right..$$

Following the location‐scale framework of Siegfried et al. (2023), we write each conditional transformation function $h_{j}(y_{j}|x)$ in terms of its own baseline transformation $h_{j}(y_{j})$, a covariate‐dependent shift term $\beta_{j}\left(x\right)$, and a covariate‐dependent scale term $\gamma_{j}\left(x\right)$:

(4) $$h_{j}\left(y_{j}|x\right)=h_{j}\left(y_{j}\right)\sqrt{\exp\left(\gamma_{j}(x)\right)}-\beta_{j}\left(x\right).$$

Here, $\gamma_{j}\left(x\right)$ and $\beta_{j}\left(x\right)$ are modeled as linear predictors $x^{⊤}\gamma_{j}$ and $x^{⊤}\beta_{j}$. The baseline transformation function $h_{j}(y_{j})$ generalizes commonly used response transformations such as log‐ or square‐root transformations, by estimating the transformation from the data, similar to but more flexible than Box‐Cox transformations (Box & Cox, 1964). Following Siegfried and Hothorn (2020), we parameterize them as a polynomial in Bernstein form $a{\left(\left\lfloor y_{j}\right\rfloor \right)}^{⊤}\vartheta_{j}$, where $\left\lfloor y_{j}\right\rfloor$ is the integer part of $y_{j}$. Monotonicity is ensured using linear constraints on the parameter vector $\vartheta_{j}$ as described in Hothorn et al. (2018). Note that $\beta_{j}\left(x\right)=E\left(h_{j}\left(Y_{j}\right)\sqrt{\exp\gamma_{j}\left(x\right)}\;X=x\right)$ represents the conditional mean of the scaled‐transformed counts.

### Covariate‐dependent correlation

The covariate‐dependent correlation matrix $\Sigma \left(x\right)$ is the central component for modeling interspecific associations. To ensure that $\Sigma \left(x\right)$ is always a valid correlation matrix during estimation, we parameterize it by means of its scaled inverse Cholesky factor

(5) $$\Lambda \left(x\right)=\left(\begin{array}{cccc} 1 & & & 0\\ \lambda_{21}\left(x\right) & 1 & & \\ \vdots & \ddots & \ddots & \\ \lambda_{J1}\left(x\right) & \ldots & \lambda_{J,J-1}\left(x\right) & 1 \end{array}\right),$$

where each unconstraint $\lambda_{ȷj}\left(x\right)$ (for ȷ>j) is expressed as a linear predictor $x^{⊤}\xi_{ȷj}$. The correlation matrix $\Sigma \left(x\right)$ is then computed via:

(6) $$\Sigma \left(x\right)=\mathrm{diag}{\left(\Lambda {\left(x\right)}^{-1}\Lambda {\left(x\right)}^{-⊤}\right)}^{-1/2}\;\Lambda {\left(x\right)}^{-1}\Lambda {\left(x\right)}^{-⊤}\times \mathrm{diag}{\left(\Lambda {\left(x\right)}^{-1}\Lambda {\left(x\right)}^{-⊤}\right)}^{-1/2},$$

where $\mathrm{diag}{\left(A\right)}^{-1/2}$ denotes the diagonal matrix with the reciprocal inverse square roots of the diagonal elements of a matrix A. This scaling ensures the diagonal elements of $\Sigma \left(x\right)$ are always equal to one, yielding a valid correlation matrix. A detailed discussion of this parameterization and scaling can be found in Hothorn (2026b).

While the parameters $\lambda_{ȷj}\left(x\right)$ have a direct interpretation (Pourahmadi, 2007), we focus on the resulting correlations $\Sigma {\left(x\right)}_{ȷj}$ as they quantify the strength of association between species ȷ and j on the latent transformed scale. To make the interpretation of those correlations agnostic of the monotone transformation $h\left(\cdot |x\right)$, we can convert them to Spearman's rank correlation coefficient ρ or Kendall's τ (Fang et al., 2002):

(7) $$\Sigma^{\left(\rho \right)}{\left(x\right)}_{ȷj}=\frac{6}{\pi }\arcsin\left(\frac{1}{2}\Sigma {\left(x\right)}_{ȷj}\right),$$

(8) $$\Sigma^{\left(\tau \right)}{\left(x\right)}_{ȷj}=\frac{2}{\pi }\arcsin\left(\Sigma {\left(x\right)}_{ȷj}\right).$$

### Likelihood inference

Count data $y_{i}\in {{\mathbb{N}}_{0}}^{J}$ can be viewed as interval‐censored information (Siegfried & Hothorn, 2020). Instead of observing an exact discrete multivariate observation $y_{i}\in {\mathbb{R}}^{J}$, a continuous random variable censored to the multidimensional interval $\left(y_{i}-1,y_{i}\right]$ is observed. Thus, for N observations $y_{i},i=1,\ldots ,N$ of a multidimensional count variable $Y={\left(Y_{1},\ldots ,Y_{J}\right)}^{⊤}$, the exact log‐likelihood in this framework is given by

(9) $$\ell \left(\theta \right)=\sum_{i=1}^{N}\log\left(\int_{h\left(y_{i}-1|x_{i}\right)}^{h\left(y_{i}|x_{i}\right)}\phi_{0,\Sigma \left(x_{i}\right)}\left(z\right)dz\right),$$

where $\phi_{0,\Sigma \left(x\right)}$ is the density of $\Phi_{0,\Sigma \left(x\right)}$ and θ contains all J marginal model parameters $\left(\gamma_{j},\beta_{j},\vartheta_{j}\right)$ and all covariance parameters of $\Lambda \left(x\right)$ (i.e., $\xi_{ȷj}$). To correctly handle zero counts, we define $h\left(y|x_{i}\right)=-\infty$ for y<0. Additionally, we handle missing values in the *j*‐th dimension of $y_{i}$ by setting the integration limits for that dimension in Equation 9 to $\left(-\infty ,\infty \right)$, rendering this dimension for this observation non‐informative without ignoring the non‐missing entries of $y_{i}$.

Maximization of the exact log‐likelihood $\ell \left(\theta \right)$ requires fast numerical procedures for the evaluation of multivariate normal integrals over hypercubes in the presence of observation‐specific, unstructured covariance matrices $\Sigma \left(x_{i}\right)$. A novel implementation of Genz' algorithm (Genz, 1992) in the *mvtnorm* R add‐on package (Hothorn, 2026a) provides functionality for the evaluation of $\ell \left(\theta \right)$ and the corresponding gradient when $\Sigma \left(x_{i}\right)$ is parameterized in terms of its Cholesky factor 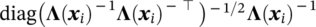 (which is simple to derive from the lower triangular matrix $\Lambda \left(x_{i}\right)$). As estimating a zero‐mean covariate‐dependent Gaussian copula is a convex optimization problem (Barratt & Boyd, 2023), this allows the application of standard solvers to the underlying optimization problem (see Hothorn, 2026b, for technical details).

The variance–covariance matrix of all model parameters can be obtained from the numerically evaluated Hessian, allowing for Wald‐type CIs. Because we estimate all model parameters jointly—rather than employing a two‐step procedure that estimates marginals prior to copula parameters—standard asymptotic theory applies, yielding asymptotically correct SEs. Uncertainty in the estimated correlation matrix $\Sigma \left(x\right)$ can be estimated via a Wald‐type parametric bootstrap, where the parameters of $\Lambda \left(x\right)$ are sampled from their asymptotic distribution.

## CASE STUDY: FISH‐EATING AQUATIC BIRDS

The development of JCTMs was motivated by the species community of three piscivorous aquatic birds, which feed on similarly sized fish within the same habitat: Great Cormorant (*Phalacrocorax carbo*), Great Crested Grebe (*Podiceps cristatus*), and Goosander (*Mergus merganser*). In Europe, these birds can play a major role in controlling the abundance of their prey fish species and, consequently, influence their own respective abundances.

### Data and study area

The analysis is based on daily counts of these bird species sampled at lake Seehamer See (Figure 1), which is located in the foothills of the Alps in southern Bavaria, about 40 km southeast of Munich. It has an area of 1.47 km^2^, with an average depth of 3.8 m (maximum 12 m). Thus, the entire water body is accessible to all three bird species. A local ornithologist, Gerhard Kinshofer, counted the three species daily between May 1, 2002 and November 13, 2016, from various locations around the lake using a spotting scope (Kinshofer et al., 2018). He was therefore able to spot most, if not all, birds on the lake. As the sampling design remained identical across all years, it can be safely assumed that any sampling error was consistent over time. The observation period comprised 5311 days. Data were missing for 0.34% of the days for some bird species, when the ornithologist was unsure whether a species was truly absent, and for 6.31% of the days for all bird species due to adverse meteorological conditions. Missing values were handled in the likelihood as described in the section *Likelihood inference*, implicitly assuming missing completely at random (MCAR). While adverse weather conditions could potentially affect bird presence or detectability, the overall low missingness rate makes it negligible for demonstrating the JCTM framework. Thus, we expect any deviations from this assumption to have only a minor influence on our results.

**FIGURE 1:**
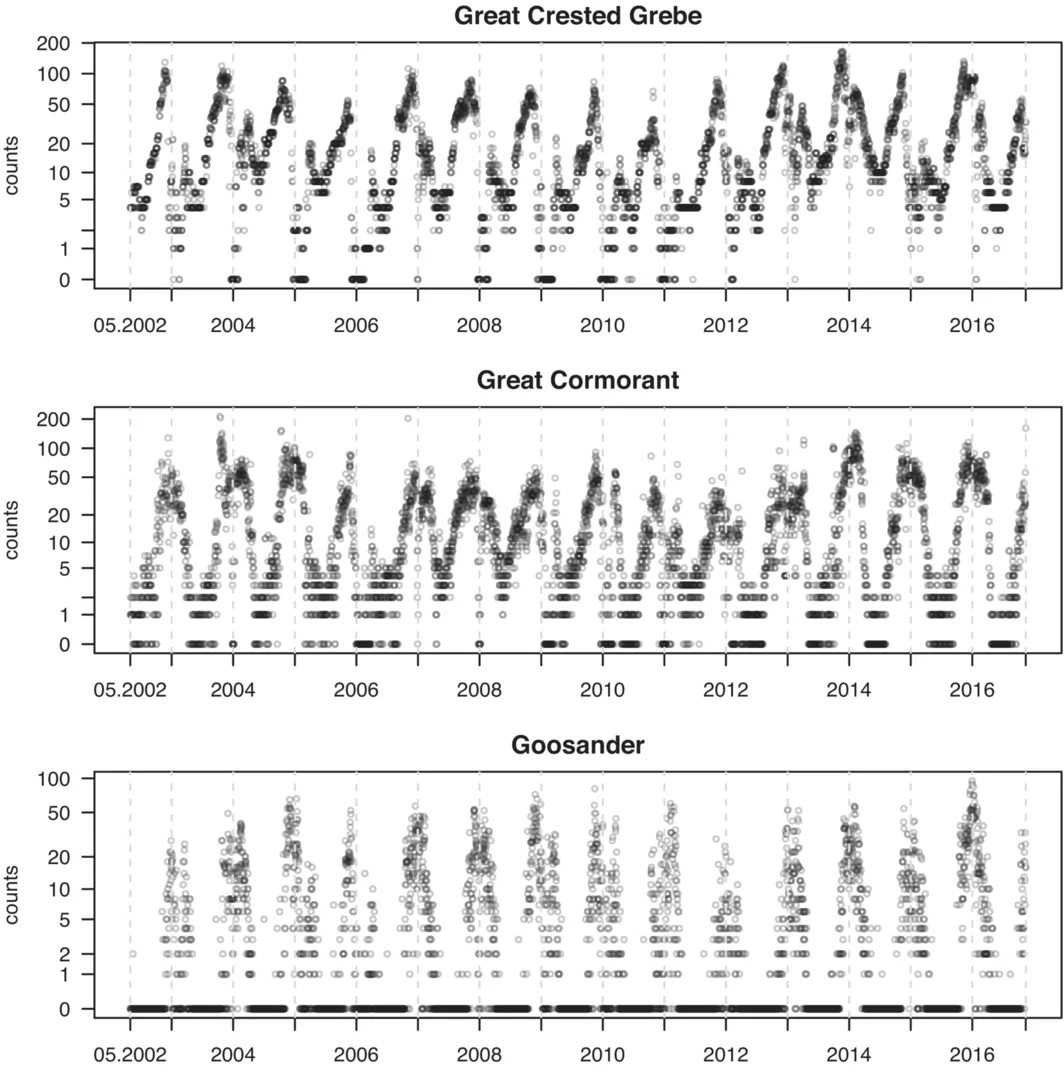
Observed count time series for the three aquatic bird species at lake Seehamer See (Germany).

The raw time series data (Figure 1) reveal clear seasonal patterns for all three species, albeit with noticeable year‐to‐year variation in counts. All three species showed lower counts during the summer months. This pattern was particularly pronounced for the Goosander, with only 13 out of 1308 observations in June, July, and August being nonzero. Conversely, the Great Crested Grebe and Great Cormorant exhibited consistent peaks in counts around November. The rate of decline following this peak differed noticeably between years, leading to high variation in the subsequent winter months (December through March). These complex distributional features—seasonal means, high zero counts, and seasonally driven variance—are precisely what our marginal SDMs are designed to capture.

### Ecological hypotheses and research questions

In the study area, the abundances of all three species in winter increased during the second half of the 20th century such that carrying capacity was reached in the 1990s (Suter, 1995). A possible explanation for the logistic growth of the winter populations is that the food resources for these piscivores were limited. All three species forage on fish of almost the same size (10–20 cm) by diving to a depth of 5 m. Thus, the three species exhibit considerable overlap in their feeding niches. Accordingly, if common resources are limiting, interspecific competition would be expected. Furthermore, the associations between the three bird species would presumably change according to seasonal variations in the abundances of the bird species (for seasonal variation of competition; see, e.g., Cecala et al., 2020; Wignall et al., 2020). Hence, in the absence of experimental intervention data, our aim was to answer the following questions: (1) How does the abundance of each species vary over the course of a year? (2) How do interspecific associations between the species vary over the course of a year? That is, what are the Spearman rank correlations between the marginal species abundances, conditional on the day‐of‐the‐year?

### Modeling bird abundances and associations using JCTMs

Using the JCTMs introduced in *Joint count transformation models*, we analyzed the joint conditional distribution of the trivariate bird‐count response $Y={\left(Y_{1},Y_{2},Y_{3}\right)}^{⊤}\in {{\mathbb{N}}_{0}}^{3}$ (1 = Great Crested Grebe, 2 = Great Cormorant, 3 = Goosander), conditional on the day‐of‐the‐year. Question (1) is addressed by the marginal SDMs (see *Marginal species distribution models*), and Question (2) is addressed by the covariate‐dependent correlations (see *Covariate‐dependent correlation*).

We specified the model components $h_{j}\left(y_{j}\right)$, $\gamma_{j}\left(x\right)$, $\beta_{j}\left(x\right)$, and $\lambda_{ȷj}\left(x\right)$ as follows. The transformation function $h_{j}\left(y_{j}\right)$ was parameterized using a polynomial in Bernstein form of order six as used for count data by Siegfried and Hothorn (2020), to significantly reduce the number of parameters while allowing substantial flexibility in the marginal models. To account for seasonal patterns in abundance and associations, we used a periodic fourth‐order Fourier basis of the day‐of‐the‐year as the linear predictor for the shift term $\beta_{j}\left(x\right)$, the scale term $\gamma_{j}\left(x\right)$, and each correlation parameter $\lambda_{ȷj}\left(x\right)$.

Figure 2 displays the estimated marginal distributions from our individual SDMs (Equation 2) as quantiles conditional on the day‐of‐the‐year, addressing our first research question. The models successfully captured the observed seasonal patterns. Notably, the inclusion of the covariate‐dependent scale term ($\gamma_{j}\left(x\right)$) allowed the model to account for the higher variability in Great Crested Grebe and Great Cormorant counts observed after the November peak. For the Goosander, the flexible baseline transformation $h_{j}\left(y_{j}\right)$ accurately modeled the high frequency of zero counts during summer while still providing a good fit to the nonzero counts observed in other seasons.

**FIGURE 2:**
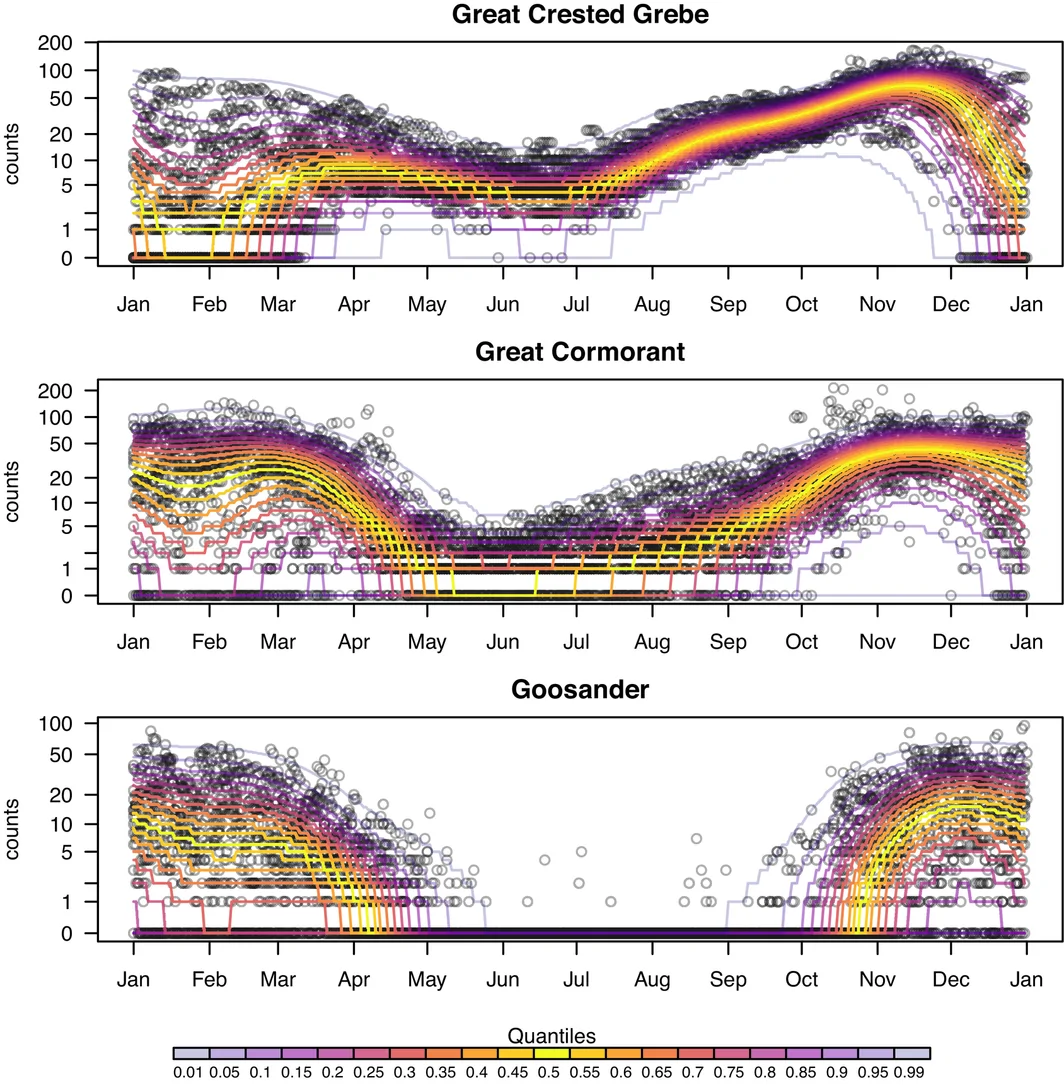
Estimated marginal distributions conditional on time of the year for the three bird species through our joint count transformation model (JCTM). Distribution is visualized via quantile curves with observed counts for all years visible as a reference.

Our maximum likelihood approach converged in 10 min on a single core. The main computational effort for our method lies in the evaluation of the exact log‐likelihood (Equation 9) and its gradient during optimization. The results detailing the covariate‐dependent interspecific correlations (addressing our second research question) are presented subsequently in *Covariate‐dependent interspecific associations* and Figure 3.

**FIGURE 3:**
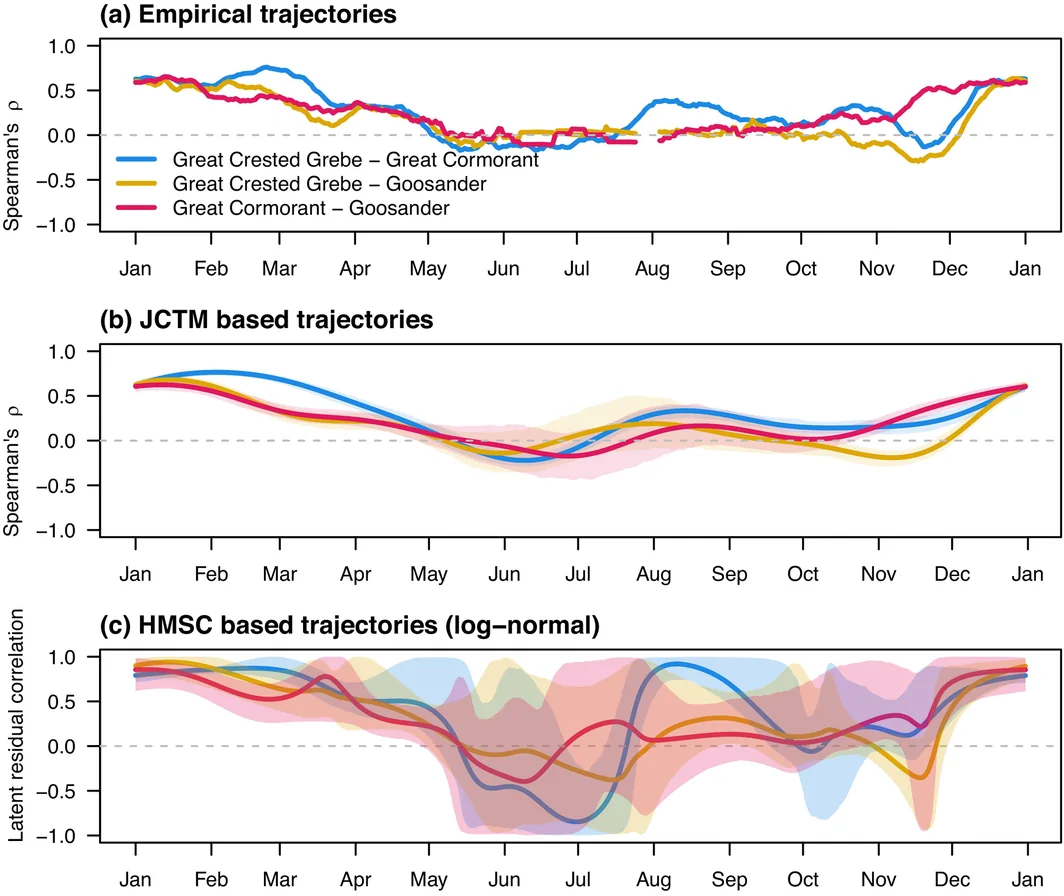
Pairwise correlation trajectories across species over one year: (a) empirically derived Spearman's rank correlations ρ using all observations within a ±10 day window across all years; (b) Joint count transformation model (JCTM)‐based Spearman's rank correlations with 95% CIs derived via parametric bootstrap; (c) the corresponding Hierarchical Modeling of Species Communities (HMSC)‐based latent residual correlations for the log‐normal model. The Markov chain Monte Carlo (MCMC) chains did not mix/converge well for the covariance parameters, so the posterior‐based 95% CIs should be interpreted with caution.

### Hierarchical Modeling of Species Communities

Furthermore, we compared JCTMs to HMSC (Ovaskainen et al., 2017), a state‐of‐the‐art framework for analyzing community data (Norberg et al., 2019). The key conceptual difference is that HMSC is a fully parametric approach that assumes a Poisson distribution for the species abundances with a log‐link function:

(10) $$Y_{j}\sim \text{Poisson}\left(\exp\left(x^{⊤}{\tilde{\beta }}_{j}+\varepsilon_{j}^{\left(\mathrm{day}\right)}+\varepsilon_{j}^{\left(\text{residual}\right)}\right)\right),$$

where species associations are captured by the covariance of a multivariate latent random effect at the day‐of‐the‐year level, $\varepsilon^{\left(\mathrm{day}\right)⊤}\sim N_{J}\left(0,\tilde{\Sigma }\left(x\right)\right)$. An extension allows these associations to depend on covariates (Tikhonov et al., 2017). The observation‐level random effect in the log‐link $\varepsilon^{\left(\text{residual}\right)}\sim N_{J}\left(0,\mathrm{diag}\left(\sigma_{1},\overset{\ldots }{,}\sigma_{J}\right)\right)$ addresses residual deviations and potential overdispersion as argued by Ovaskainen and Abrego (2020). Even though we use the same linear predictors for the location and covariance terms as in JCTMs, we expect $x^{⊤}{\tilde{\beta }}_{j}$ and $\tilde{\Sigma }\left(x\right)$ to differ, as HMSC only models the conditional means of Poisson distributions. In contrast, JCTMs learn the shape of the entire conditional distribution from the data, avoiding strong distributional assumptions.

In stark contrast to our approach, the HMSC model was fitted using Bayesian MCMC estimation (Tikhonov et al., 2025) and required 10 days and 5 h to generate 150,000 MCMC samples (including 100,000 burn‐in) per chain, running four chains in parallel on four cores. Moreover, despite this immense computational overhead, convergence diagnostics for the covariance parameters indicated poor mixing: the median univariate potential scale reduction factor was 5.53, the multivariate potential scale reduction factor was 115.15, and the effective sample sizes were severely low (median 341, minimum 73) (Plummer et al., 2024).

### Covariate‐dependent interspecific associations

The main motivation for JCTMs was identifying covariate‐dependent interspecies dependencies, addressing our second research question. Before presenting the two model‐based approaches, we first explore these dynamics empirically. For each day‐of‐the‐year, we computed the rank correlation coefficient (Spearman's ρ) for each pair of species using all observations across all years within a ±10‐day window, to obtain a smoother estimate. Figure 3a displays the resulting correlation curves, suggesting higher probabilities of co‐occurrences in the colder months and uncorrelated species counts in the warmer months. This empirical window‐based approach provides an intuitive initial perspective on the seasonal dependence of interspecific associations. However, the resulting estimates are inherently noisy and lack an uncertainty quantification.

The time‐dependent Spearman's rank correlations extracted from our model are presented in Figure 3b. Our smooth, model‐based correlation curves closely resembled the patterns observed in the empirical correlation trajectories in Figure 3a. In particular, the model confirmed the high positive correlations between all three bird species during the winter months. We also observed periods of moderate negative correlation, such as between the Great Crested Grebe and Great Cormorant in June, and between the Great Cormorant and Goosander in November. As we observed relevant autocorrelation in the model residuals (not shown), the parametric bootstrap CIs are likely too narrow. However, we expect them to still give a qualitative impression of the relative uncertainty in the estimates. And indeed, the uncertainty was highest for correlations involving the Goosander during the summer months, a direct consequence of the sparse nonzero counts in that period. Consequently, we interpreted the correlation estimates in these high‐uncertainty regions with caution.

For comparison, we fitted the log‐normal HMSC variant with covariate‐dependent correlations. The resulting correlation curves shown in Figure 3c were qualitatively similar to those from our JCTM (Figure 3b). An exact match is not expected, given the conceptual differences: HMSC models latent residual correlations based on a strict Poisson assumption for the counts, whereas our model estimates the rank correlation from flexibly learned, distribution‐free marginals.

## DISCUSSION

JCTMs provide a new perspective on joint models for species distributions, utilizing flexible distribution‐free marginal SDMs combined with a covariate‐dependent copula, in a unified maximum likelihood framework for count data. In this application, we focused on seasonality as the only covariate of interest influencing both marginal abundances and pairwise correlations. However, in general, the framework can be extended to include other covariates x, such as environmental factors, physical habitat variables, meteorological conditions, and spatiotemporal dynamics.

In contrast to common MSDMs and JSDMs (mostly MGLMMs and GLLVMs), JCTMs do not make any assumptions about a parametric distribution of the counts. Instead, the distribution is estimated in a semi‐parametric way. This avoids the need to separately consider overdispersion or high zero counts and extends the modeling to situations where common parametric distributions like the Poisson or Negative Binomial are not justified. Additionally, JCTMs represent the community aspect (i.e., measures of dependence) not by a multivariate random residual term in the linear predictor, but rather by pairwise Pearson's correlations between $h_{j}\left(y_{j}|x\right)$ and $h_{ȷ}\left(y_{ȷ}|x\right)$ for species j and ȷ, which can be understood as Spearman's rank correlations between species on the original scale of the counts as shown in Figure 3a,b. Common MSDMs and JSDMs do not provide such rank‐based measures of dependence, but come with Pearson's correlations of random effects on the scale of the link function with no intuitive interpretation. Moreover, when interpreting fixed effects, JCTMs do not require conditioning on random effects, as is the case when the commonly fixed/random effects dichotomy is applied. Consequently, JCTMs allow us to answer a broader class of research questions that simultaneously involve marginal SDMs and the relationship between different species depending on covariates x. Furthermore, modeling correlations dependent on covariates accommodates covariate‐induced variations in species associations in addition to possible covariate‐driven trends in marginal abundances.

In the relatively simple models for aquatic bird associations presented herein, the marginal abundances of the three fish‐eating bird species were shown to vary systematically over the course of a year (hypothesis 1, reflecting different habitat requirements particularly during the breeding period in summer), with moderate positive correlations determined in winter and early spring, and small and highly variable correlations during the rest of the year (hypothesis 2). However, as highlighted by Poggiato et al. (2021), residual correlations in phenomenological JSDMs capture a combination of true biotic interactions and shared responses to missing environmental covariates. Therefore, the positive winter associations observed in our model are potentially driven by shared responses to unmeasured environmental factors—such as seasonal fish aggregations or favorable climatic conditions—rather than direct mutualism. Conversely, these strong shared environmental preferences might actually be masking underlying competitive dynamics.

JCTMs are currently limited in several ways. With covariate‐dependent inverse Cholesky factor $\Lambda \left(x\right)$, the model is not as lean on assumptions as a model with constant correlations, that is, constant parameters in Λ. Concretely, the parameter estimates resulting from the joint model might depend on the order in which the marginal models are specified (Barratt & Boyd, 2023). This effect can be counterbalanced by accounting for sufficient flexibility in the marginal models. Models with covariate‐dependent correlations $\Sigma \left(x\right)$ should be estimated for different permutations of the species in a sensitivity analysis. In general, a strong dependence of the parameter estimates on the order of species indicates a severe lack of model fit.

Alternative interpretations of $\beta_{j}\left(x\right)$, such as odds ratios and hazard ratios, are discussed in Hothorn et al. (2018) and require adapting Equation 1 according to section 2.6 in Klein et al. (2022). Model estimation is implemented in *tram* for a range of alternatives. Stratification can be achieved by estimating the baseline transformation function $h_{j}$ in Equation 4 for each stratum separately. This and more complex interactions between x and $y_{j}$ are further discussed in Siegfried et al. (2023). Complex models can be specified for the marginal shift terms $\beta_{j}\left(x\right)$ and for components of the Cholesky factor $\Lambda \left(x\right)$, for example by including either nonlinear or lagging effects of explanatory environmental variables or terms capturing spatiotemporal trends for correlated observations. However, in such cases, the likelihood approach proposed here requires supplementation with appropriate smoothness constraints. A mixed version of univariate transformation models that is also applicable to univariate count transformation models was recently proposed (Tamási, 2025) and can handle spatially or otherwise correlated data. However, the technical challenges associated with mixed transformation models on the one hand and JCTMs on the other clearly demonstrate that significant improvements in this direction will require substantial research efforts. With respect to nonlinear habitat effects, a pragmatic approach might be to estimate marginal SDMs separately for each species, thus allowing nonlinear associations with environmental variables. The joint models could then be fitted with the same functional form as that of the marginal shift terms $\beta_{j}\left(x\right)$. If the goal is proper statistical inference of model parameters, one should check for conditional independence, for example, by inspecting spatiotemporal residual autocorrelation. The presence of such autocorrelation would indicate that the assumption is violated and reinforces the need to extend the model to a mixed‐effects version, as discussed previously.

In a tailored simulation study (see Appendix S1), we evaluated how estimation accuracy and computation time change with the number of species J, the sample size N, and M, the number of Monte Carlo samples used to approximate the log‐likelihood in Equation 9. We observed consistent estimation in N and a computational complexity of $O\left(\mathrm{MN}\right)$, which is expected given the form of the log‐likelihood. However, the computational burden scales roughly exponentially with J. This is expected given that the number of parameters in $\Lambda \left(x\right)$ grows quadratically with J and that more Monte Carlo samples are required to maintain precision when the dimension increases. While computationally challenging, this simulation shows that joint maximum likelihood estimation for JCTMs is now feasible for communities with up to 10 species, due to the availability of the corresponding score function (see Hothorn, 2026a, for computational details). For J>15, there exist several approaches to reduce the computational burden. One option is to replace the joint estimation with a two‐step procedure, where the marginal SDMs are estimated separately and the copula parameters are estimated in a second step. Another option is to treat the counts as a continuous variable and apply the continuous version introduced by Klein et al. (2022). However, as our simulation demonstrates, this comes at the severe cost of accuracy and is highly detrimental for sparse, low‐count ecological data (such as our Goosander observations). If the covariate‐dependence of the correlation structure is not of interest, one could also consider JSDMs that use a rank‐reduction for the correlation structure (e.g., McGillycuddy et al., 2025).

JCTMs are potentially complex and cannot be viewed as extensions of established JSDMs or MSDMs. Model interpretation requires an understanding of latent, transformed scales, especially on the marginal scale. Nevertheless, we demonstrated that interesting insights into the interplay between species can be obtained from a simple JCTM.

### Computational details

All computations were performed using R version 4.6.0 (R Core Team, 2026). Joint count transformation models are implemented in the *tram* add‐on package (Hothorn et al., 2026). Models and figures from the manuscript and the simulation study can be reproduced with the *cotram* package (Siegfried et al., 2026) using the code below.


library
(
"cotram"
)


demo
(
"aquabirds"
,
 
package
 
=
 
"cotram"
)


source
(
system.file
(
"simulation"
,
 
"SIM‐JCTM.R"
,
 
package
 
=
 
"cotram"
))





The competitor model HMSC was fitted with the package *Hmsc* (Tikhonov et al., 2025).

## AUTHOR CONTRIBUTIONS

Lukas Graz, Luisa Barbanti, and Torsten Hothorn developed the models and maximum likelihood estimation procedures. Roland Brandl contributed the aquatic bird model system. All authors analyzed and interpreted this data, drafted, and finally revised the manuscript.

## CONFLICT OF INTEREST STATEMENT

The authors declare no conflicts of interest.

## Supporting information


Appendix S1.

## Data Availability

The materials to reproduce this work are provided in Siegfried et al. (2026) in the *cotram* R‐package on CRAN at https://doi.org/10.32614/CRAN.package.cotram.
