# Supplementary material for: Joint count transformation models with covariate‐dependent correlations
Source: Ecology. 2026 Oct 12;107(10):e70522. doi: 10.1002/ecy.70522 (PMC13656892; doi:10.1002/ecy.70522)
Supplement: Supplementary file 1 [file ECY-107-e70522-s001.pdf]

Appendix S1

# Joint count transformation models with covariate-dependent correlations

Lukas Graz
Luisa Barbanti
Roland Brandl
Torsten Hothorn

Ecology

Appendix S1: Simulation study

The empirical case study in Section 3 is intentionally simple, involving only three species. To assess how JCTMs behave beyond this setting, we conducted a simulation study with increasing dimensionality and sample size. Our goal is to quantify how estimation accuracy and computation time change with the number of species J, the number of observations N, and M, the number of Monte Carlo samples used to approximate the log-likelihood in Equation 9. Furthermore, we study how this differs between datasets with low and high counts, as the continuous (later abbreviated as cts.) approximation $\ell_{\text{cts.}}$ from Klein et al. (2022) is expected to be less severe for higher counts. By varying J, we assess scalability in higher dimensions; by varying N, we assess finite-sample bias and consistency.

Performance metrics

We evaluate the computation time and the accuracy of the estimated non-transformation parameters $\boldsymbol{\psi} = (\boldsymbol{\beta}, \boldsymbol{\xi})$, i.e., the marginal shift parameters and all covariance parameters of $\boldsymbol{\Lambda}(\boldsymbol{x})$ used for the covariate-dependent correlation structure $\boldsymbol{\Sigma}(\boldsymbol{x})$. First, we compute starting values $\hat{\boldsymbol{\psi}}_{\text{cts.}}$ using the continuous approximation and record the computation time $t_{\text{cts.}}$ for M samples. Then, using the Monte Carlo approximation, we compute $\hat{\boldsymbol{\psi}}_M$ and record the total computation time t_M in seconds.

To assess the accuracy of the shift parameters, we compute the mean absolute error of estimating $\boldsymbol{\beta}$:

$$\text{MAE}_{\boldsymbol{\beta}} = \text{mean}\left(\left|\boldsymbol{\beta} - \hat{\boldsymbol{\beta}}\right|\right),$$

For the covariate-dependent correlation matrix, we evaluate the MAE of its off-diagonal elements for $x \in \{0, 1\}$, evaluating the error at the boundaries (or endpoints) of the covariate space to capture the maximum potential divergence in the correlation trajectories:

$$\text{MAE}_{\boldsymbol{\Sigma}} = \text{mean}_{i<j}\left(\left|(\boldsymbol{\Sigma}(0)_{ij} - \hat{\boldsymbol{\Sigma}}(0)_{ij})\right| + \left|(\boldsymbol{\Sigma}(1)_{ij} - \hat{\boldsymbol{\Sigma}}(1)_{ij})\right|\right).$$

To evaluate the accuracy of the Monte Carlo approximation, we compare the log-likelihood ℓ_{M} from Equation 9, approximated using M Monte Carlo samples, with the analogous high-precision baseline ℓ_{10^6} computed with $M = 10^6$ samples. To provide a baseline for this approximation, we compare it to the continuous log-likelihood $\ell_{\text{cts.}}$, which ignores the discrete nature of the counts. For these two comparisons, we inspect the absolute relative differences in log-likelihoods:

$$\Delta L_{\text{rel,M}} := \frac{|\ell_{\text{M}} - \ell_{10^6}|}{|\ell_{10^6}|} \quad \text{and} \quad \Delta L_{\text{rel,cts.}} := \frac{|\ell_{\text{cts.}} - \ell_{10^6}|}{|\ell_{10^6}|},$$

respectively. Additionally, as a measure of how well the Monte Carlo approximation facilitates the overall optimization, we report the mean absolute gradient of the log-likelihood for $\boldsymbol{\psi}$, evaluated using M Monte Carlo samples:

$$\text{MAG}_{M,\boldsymbol{\psi}} = \text{mean}\left(\left|\nabla_{\boldsymbol{\psi}} \ell_{\text{M}}(\boldsymbol{\psi})\right|\right).$$

In particular, we evaluate the three variants: $\text{MAG}_{M,\hat{\boldsymbol{\psi}}_M}$ capturing how well the optimizer managed to find the optimum of ℓ_M, $\text{MAG}_{10^6,\hat{\boldsymbol{\psi}}_M}$ capturing how well the Monte Carlo approximation

performes in comparison to a high precision baseline, and $\text{MAG}_{10^6,\hat{\psi}_{\text{cts.}}}$ capturing how well the continuous approximation performs in comparison to the same high-precision baseline.

Data generating process

As in Section 2.4 we treat Y as a censored version of $Y^\star \in \mathbb{R}^J$, for which we set up a parametric model. We sample from this model and obtain the corresponding counts by applying the ceiling function to each dimension, $Y = \lceil Y^\star \rceil$. To keep the setting simple we omit the scale term (i.e., $\boldsymbol{\gamma}_j(x) = 1$) and restrict the simulation to a single covariate $\boldsymbol{x} \sim \text{U}([0,1]^N)$, influencing the covariate-dependent correlation and the j-th marginal shift effects $\boldsymbol{\beta}_j(x) = \beta_j x$, where $\boldsymbol{\beta} \sim \text{U}([-1,1]^J)$. For the transformation function $h_j(y)$ we use the inverse of a chi-square cdf with df_j degrees of freedom. For the low-count datasets, we sample $\text{df} \sim \text{U}(\{3,4,\ldots,9\}^J)$, whereas for the high-count datasets we sample $\text{df} \sim \text{U}(\{30,31,\ldots,50\}^J)$. For the covariate-dependent correlation we define $\boldsymbol{\Sigma}_0, \boldsymbol{\Sigma}_1$ to be standardized independent samples from a Wishart distribution with an identity scale matrix and $J_{\max}$ degrees of freedom, and $\boldsymbol{\Lambda}_0, \boldsymbol{\Lambda}_1$ their respective scaled inverse Cholesky factors as in Equation 6. The desired $\boldsymbol{\Sigma}(x)$ is then computed via Equation 6 using $\boldsymbol{\Lambda}(x) = \boldsymbol{\Lambda}_0 + x(\boldsymbol{\Lambda}_1 - \boldsymbol{\Lambda}_0)$. The above quantities are sampled once and then kept fixed for all simulation scenarios and repetitions; for a scenario with $J < J_{\max}$ we use the leading $J \times J$ submatrices. Sampling the i-th observation $Y^{(i)}$ of a new dataset thus requires sampling $\boldsymbol{U} \sim \text{U}([0,1]^J)$, and computing $Y_j^{(i)} = \lceil F_{Y^\star}^{-1}(\boldsymbol{U})_j \rceil = \lceil h_j^{-1}(\Phi_{\boldsymbol{0},\Sigma(x_i)}^{-1}(\boldsymbol{U})_j + \beta_j x_i) \rceil$. In this manner, we generate 15 datasets with $J_{\max} = 15$ and $N_{\max} = 10000$, and later take the subset containing the first N observations and the first J species for each scenario. For the low-count datasets, the 0%, 25%, 50%, 75%, and 100% quantiles across all datasets are 1, 3, 6, 9, and 49, respectively. For the high-count datasets, these quantiles are 7, 33, 41, 48, and 112, respectively.

Results and discussion

To investigate these parameters, we evaluated the scenarios detailed in the figures below. Each data point in these plots corresponds to the median over 15 repetitions of the simulation, where we computed the aforementioned performance measures and computation times for each repetition.

Consistency with increasing sample size (Figure S1). Both the accuracy of the estimated shift parameters ($\text{MAE}_{\boldsymbol{\beta}}$) and the correlation structure ($\text{MAE}_{\boldsymbol{\Sigma}}$) improve consistently with increasing N for both low- and high-count settings, empirically confirming that the implementation of the theoretically consistent joint maximum likelihood estimator converges to the unknown model parameters. Computation time scales linearly with N, as expected from the form of the log-likelihood in Equation 9.

Accuracy of the Monte Carlo approximation (Figure S2). Computation time scales linearly with M, as expected. The relative log-likelihood difference $\Delta\ell_{\text{rel,M}}$ and the mean absolute gradient $\text{MAG}_{10^6,\hat{\psi}_M}$ both decrease with increasing M, confirming that the Monte Carlo approximation improves with more samples. Across all settings, the Monte Carlo approximation is substantially more accurate than the continuous approximation $\ell_{\text{cts.}}$, with the gap being particularly pronounced for low-count data, where treating discrete counts as continuous is least appropriate. Furthermore,

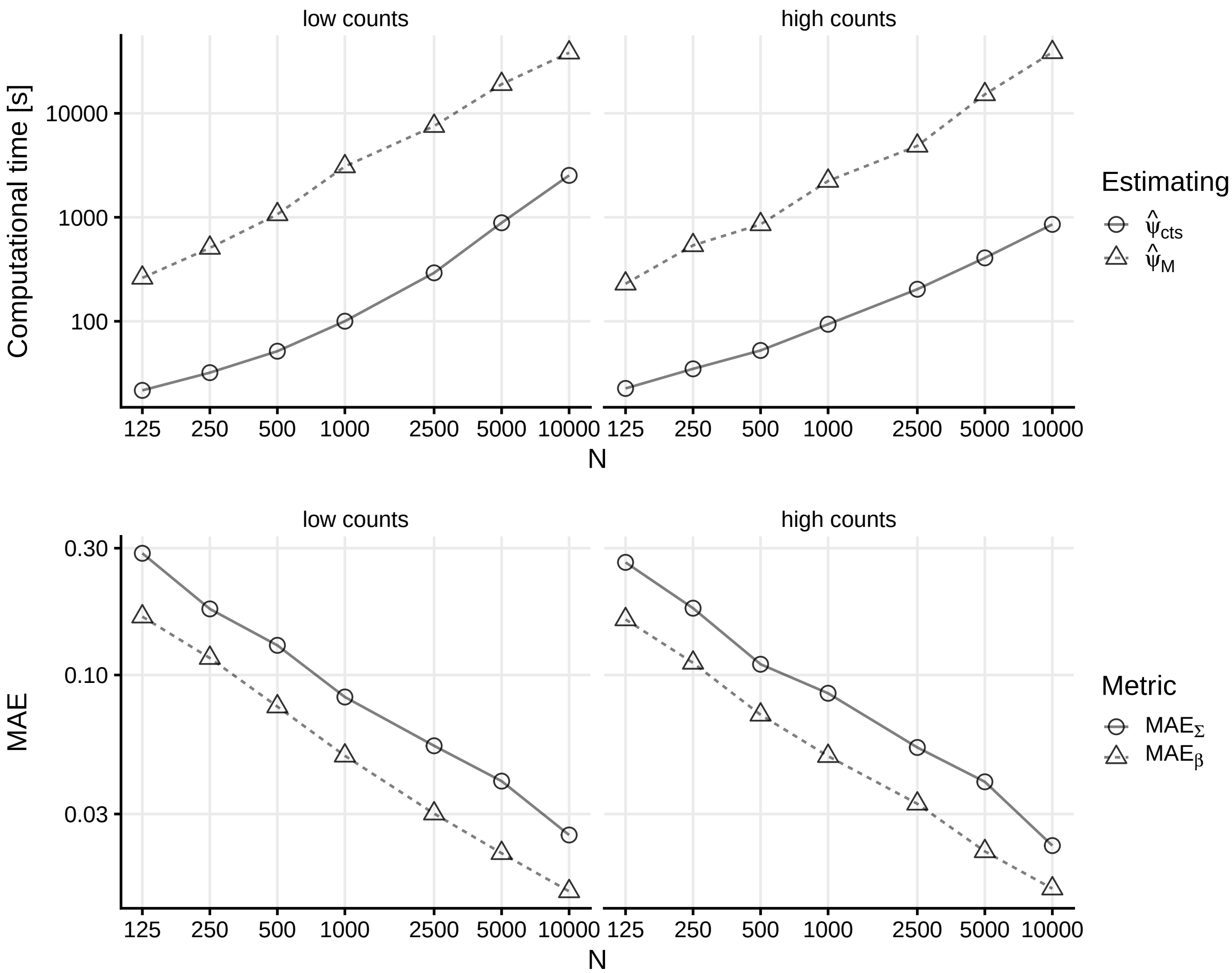

Figure S1: Consistency with increasing sample size N (fixed $J = 10$, $M = 500$).

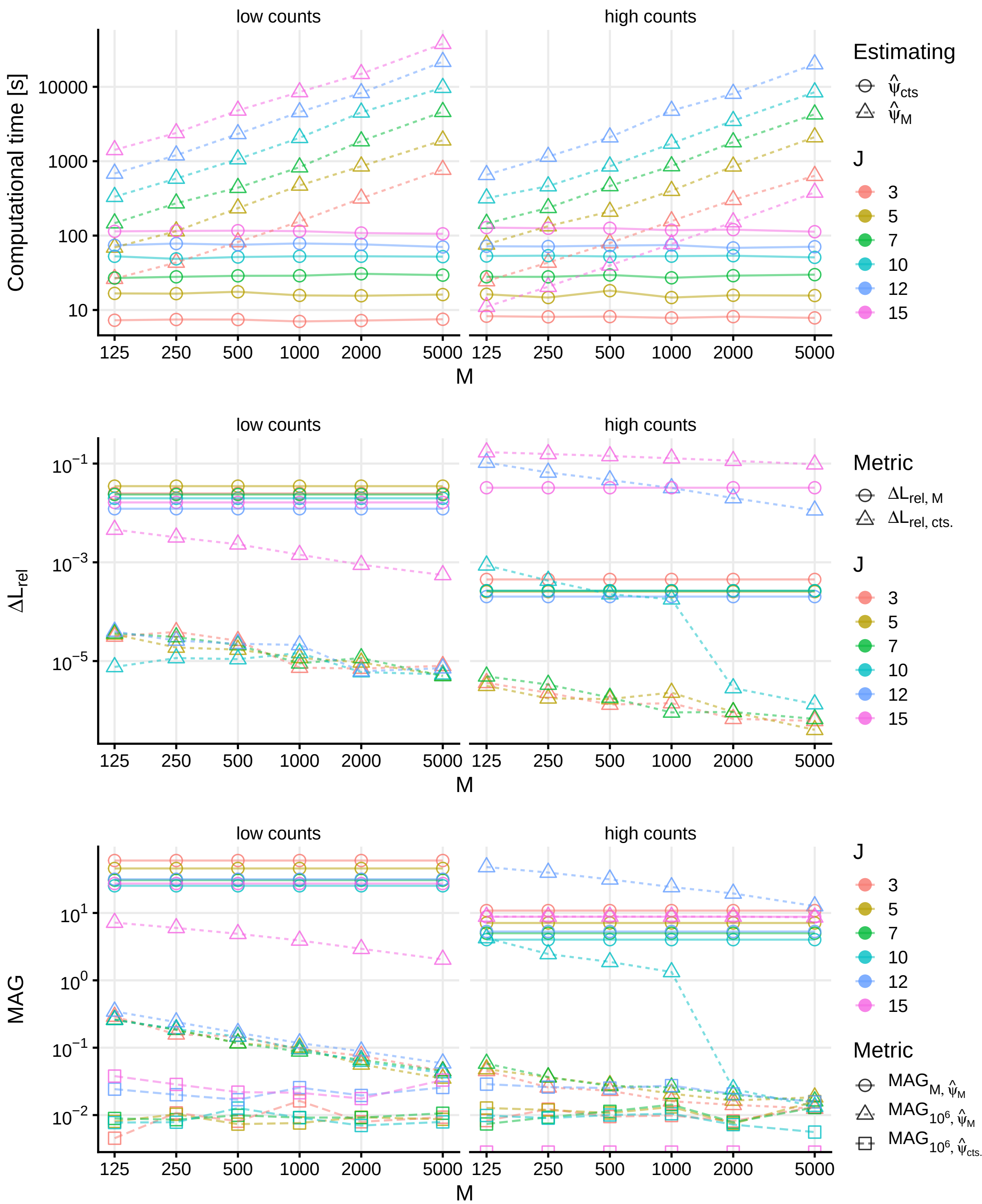

Figure S2: Accuracy of the Monte Carlo approximation with increasing number of samples M and number of species J (fixed $N = 500$). *Note: Sudden drops in computation time (t_M) or anomalous gradient trajectories for high-count datasets with $J \geq 12$ indicate optimizer convergence failures.*

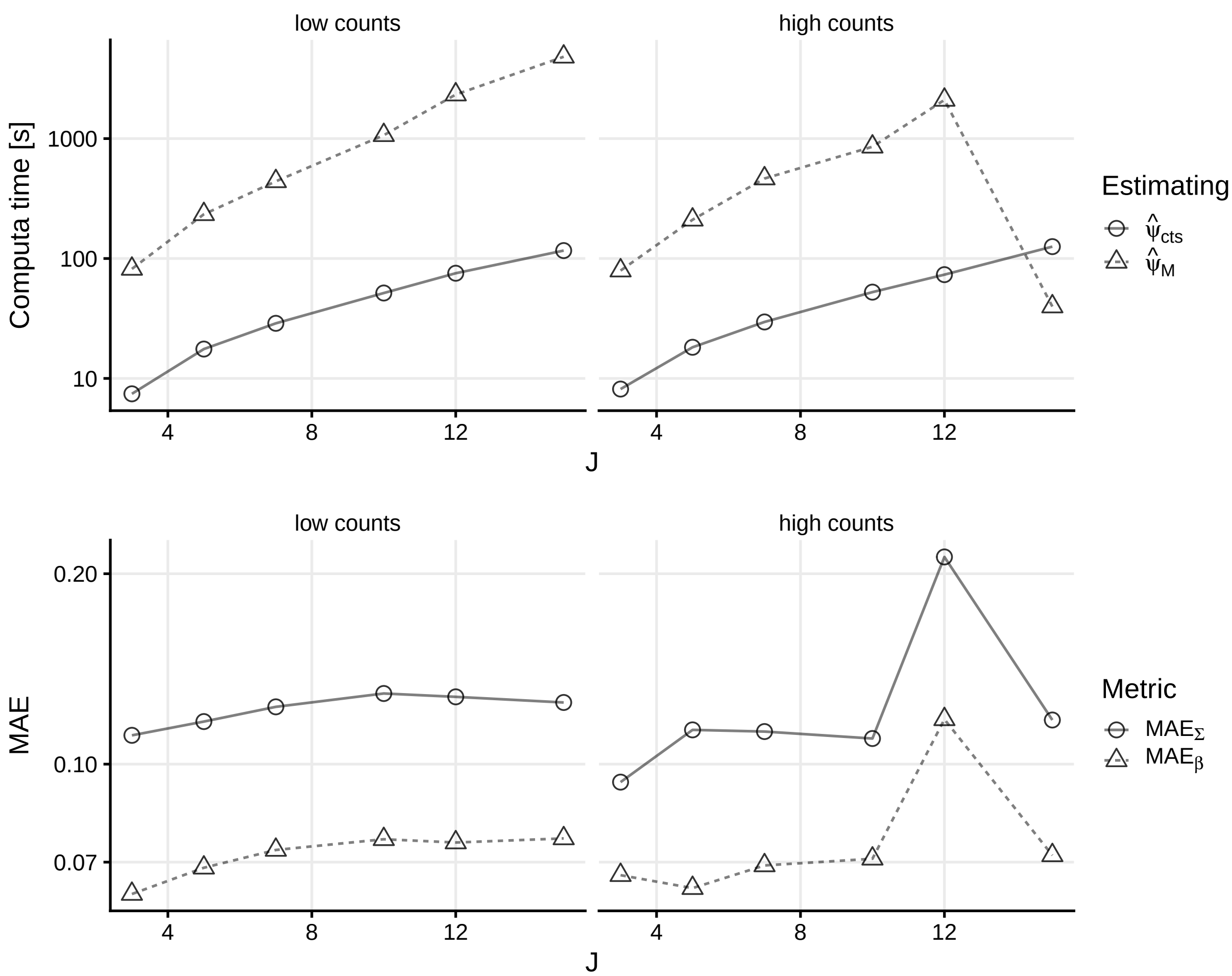

Figure S3: Scalability with increasing number of species J (fixed $N = 500$, $M = 500$). *Note: Anomalies in computation time and accuracy metrics for high-count datasets with* $J \geq 12$ *indicate optimizer convergence failures.*

comparing the trajectories for $\text{MAG}_{10^6,\hat{\psi}_M}$ and $\text{MAG}_{10^6,\hat{\psi}_{\text{cts.}}}$ in Figure S2 confirms that the continuous approximation is less severe for high-count data. This highlights the importance of using the exact discrete likelihood for count data, and demonstrates that the Monte Carlo approximation can achieve this at a manageable computational cost. The trajectory for $\text{MAG}_{10^6,\hat{\psi}_M}$ shows that optimization of ℓ_M was successful, and this quantity could be further reduced by adapting corresponding convergence tolerance parameters at the cost of increased computation time.

Scalability with increasing number of species (Figure S3). Even when holding M constant, the computational burden scales roughly exponentially with J. This exponential scaling becomes even more apparent if M must be increased to maintain precision in higher dimensions.

We conclude that joint maximum likelihood estimation of JCTMs is reasonably fast and accurate for $J \leq 10$. For $J \geq 15$, the computational burden quickly becomes prohibitive and the accuracy decreases. Note that for high-count datasets with $J \geq 12$ (Figures S2 and S3), the sudden drops in computation time and anomalies in the accuracy metrics indicate numerical underflow and optimizer convergence failures, rather than true efficiency gains. Alternatives such as a two-step procedure—estimating marginal SDMs first and the copula parameters second—or the continuous approximation $\ell_{\text{cts.}}$ may be the only practical options, at the cost of less accurate standard errors or a less appropriate likelihood, respectively. For applications where covariate-dependent correlations are not the primary interest, dimension reduction approaches such as those proposed by McGillycuddy et al. (2025) offer an additional avenue for scaling to larger communities.

References

Nadja Klein, Torsten Hothorn, Luisa Barbanti, and Thomas Kneib. Multivariate conditional transformation models. *Scandinavian Journal of Statistics*, 49(1):116–142, 2022. doi: https://doi.org/10.1111/sjos.12501. URL https://onlinelibrary.wiley.com/doi/abs/10.1111/sjos.12501.

Maeve McGillycuddy, Gordana Popovic, Benjamin M. Bolker, and David I. Warton. Parsimoniously fitting large multivariate random effects in **glmmTMB**. *Journal of Statistical Software*, 112:1–19, 2025. doi: 10.18637/jss.v112.i01.
